# Upper Limbs Movement Frequency: Connection to Swimming Performance and Kinematics

**DOI:** 10.3390/jfmk11020140

**Published:** 2026-03-27

**Authors:** Konstantinos Papadopoulos, Gavriil G. Arsoniadis, Argyris G. Toubekis

**Affiliations:** 1 Division of Aquatic Sports, School of Physical Education and Sports Science, National and Kapodistrian University of Athens, 17237 Athens, Greece; konspapadop@phed.uoa.gr (K.P.); garsoniadis@phed.uoa.gr (G.G.A.); 2 Sports Performance Laboratory, School of Physical Education and Sports Science, National and Kapodistrian University of Athens, 17237 Athens, Greece

**Keywords:** stroke rate, movement speed, swimming performance

## Abstract

**Background**: Stroke rate (SR) is a critical determinant of swimming speed and performance; however, the relationship between upper-limb movement frequency assessed on land and SR in water remains unclear. **Methods**: This preliminary study examined the association between dry-land upper-limb movement frequency and in-water kinematics in ten male competitive swimmers (15.23 ± 1.06 years). Dry-land upper-limb movement frequency was evaluated through two maximum-effort trials consisting of 10–12 circular arm movements performed under a straight-arm condition (STR_SR_) and a swimming-technique-simulated bend-arm condition (TECH_SR_). All trials were video-recorded for analysis. In-water testing included a maximum-effort 50 m sprint and 8–12 × 25 m progressively increasing speed tests to elicit maximum swimming speed and maximum SR. SR, swimming speed, stroke length, and stroke index were calculated for all trials. **Results**: No relationship was observed between dry-land upper-limb movement frequency and SR of the 50 m test (*p* > 0.05). However, the percentage difference between STR_SR_ and maximum SR was associated with stroke index at maximum speed (*r* = −0.66, *p* = 0.04), maximum SR (*r* = −0.65, *p* = 0.04), and swimming speed at maximum SR (*r* = −0.72, *p* = 0.01) οf the 8–12 × 25 test. Similarly, TECH_SR_ was correlated with stroke index at maximum speed, SR, and stroke length in the 8–12 × 25 test (*r* = −0.67 to −0.71, *p* = 0.01). **Conclusions**: These findings suggest that faster and more efficient swimmers exhibit a greater difference between their maximum dry-land movement frequency in upper limbs and the SR adopted in water, allowing a greater potential to adjust in a more comfortable and submaximal manner in water movements.

## 1. Introduction

The frequency of body segments represents a fundamental component of human motor behavior and underpins performance across a wide range of daily and sporting activities [1]. The movement frequency of upper limbs is strongly influenced by task complexity, with more complex actions requiring longer completion times and more elaborate motor control strategies [1]. Moreover, variations in movement frequency are accompanied by distinct neuromuscular activation patterns, reflecting different demands on motor-unit recruitment and coordination [2]. In sport, movement frequency (or movement speed) is considered a key determinant of performance, particularly in disciplines that require rapid, cyclic, or explosive actions [3]. In dry-land environments, the frequency of body-segment movements can be assessed with relatively high precision, enabling detailed evaluation of both efficiency and execution accuracy.

Moreover, dry-land assessments of upper-limb or lower-limb movement speed are commonly employed to evaluate neuromuscular readiness and monitor training adaptations [4,5]. These tests (e.g., pull-ups, squat jumps) are often assumed to reflect a swimmer’s potential to generate rapid, cyclic movements during swimming [5]. However, the extent to which such land-based movement capacities translate into aquatic stroke behavior remains insufficiently understood.

In swimming, the frequency of cyclic upper-limb motion, commonly expressed as stroke rate (SR), is one of the primary kinematic variables associated with swimming speed and performance. SR is defined as the number of complete arm cycles performed per time unit and, together with stroke length (SL), determines swimming speed [6,7,8]. Increasing SR generally results in higher swimming speed up to a maximum threshold, beyond which further increases may compromise coordination and reduce propulsion efficiency, ultimately impairing performance [8,9]. Consequently, optimal swimming performance is achieved by balancing SR and SL, rather than by maximizing each variable independently [9,10].

Higher SR values may contribute to reduced drag and shorter non-propulsive phases of the stroke cycle, provided that technique is preserved [8,11]. Anthropometric characteristics also influence SR regulation; swimmers with greater stature, body mass, and arm span often demonstrate longer stroke lengths and, in some cases, lower SRs [12,13,14]. However, evidence also suggests that faster swimmers may combine favorable anthropometrics with relatively high SRs, particularly in sprint events [8,15,16].

Unlike movements performed on dry land, swimming actions are executed in a highly resistive, coordination-dependent environment [17,18]. The mechanical demands of water require not only movement speed but also precise timing and force application to sustain propulsion. Consequently, the ability to perform rapid limb movements on land may not directly translate to effective stroke execution in water, highlighting a potential dissociation between neuromuscular capacity and performance [6].

Beyond biomechanical and anthropometric determinants, SR regulation can also be interpreted within broader motor-control and locomotion frameworks. According to the speed-accuracy trade-off principle [19], increases in movement frequency are often accompanied by reductions in coordination stability and force precision, particularly in cyclic tasks. Evidence from endurance locomotion models indicates that athletes rarely operate at their maximum possible frequency; instead, they self-select an optimal frequency that balances mechanical output, metabolic cost, and coordinative control, as demonstrated in cycling cadence selection and rowing stroke-rate regulation [20]. Similarly, sprint-running literature suggests the existence of a “neuromuscular reserve,” in which maximal stride frequency exceeds that used at optimal performance velocity [21]. Translating these principles to swimming, maximal dry-land upper-limb turnover may represent a neuromuscular ceiling, whereas in-water SR likely reflects a task-specific optimal solution constrained by hydrodynamics, coordination demands, and propulsive effectiveness.

Despite the established importance of SR in swimming, the relationship between upper-limb movement frequency in aquatic and dry-land environments has not been systematically examined. Specifically, it remains unclear whether the capacity to perform rapid upper-limb movements out of the water relates to in-water SR behavior and swimming performance. From a practical perspective, understanding the relationship between dry-land upper-limb movement frequency and in-water SR may help coaches interpret the relevance of dry-land frequency assessments and plan specific exercises to improve swimming performance. The last could inform training design, particularly when balancing dry-land neuromuscular work with in-water technical and kinematic objectives. Examining both dryland and in-water variables enables a more comprehensive understanding of the relationship between neuromuscular ability and its expression within the specific mechanical and coordinative constraints of swimming. This comparison offers insights into how well dryland movement potential translates into effective stroke control in water.

In practical settings, swimmers often perform dry-land exercises and technique-oriented drills (e.g., resisted arm actions, coordination drills, and high-tempo upper-limb movements) with the assumption that these will enhance SR and stroke length in water [5]. Additionally, establishing practical reference values can help coaches interpret the relationship between upper limb dryland movement frequency and in-water stroke behavior. However, the extent to which these practices lead to effective in-water stroke regulation remains unclear. Therefore, the aim of the present study was to investigate the relationship between upper-limb movement frequency measured out of the water, SR during swimming, and front-crawl performance. It was hypothesized that upper-limb movement frequency evaluated out of the water would be positively associated with SR and swimming performance during front-crawl swimming.

## 2. Materials and Methods

### 2.1. Participants

Ten male highly trained national-level swimmers (tier 3) [22] participated in this preliminary and pilot study. All swimmers competed in their national age-group championships during the competitive season (2024 to 2025). Table 1 displays the swimmer’s characteristics. A priori power analysis using G*Power 3.1 statistical package indicated a required sample size *n* = 10, given error probability (0.05), power (0.68), and a medium effect size (Cohen’s f: 0.40, d = 0.85, partial eta squared: 0.14) [23]. Considering the sample size in the present study (*n* = 10) and a corresponding minimum partial eta squared for the main effects (η^2^ = 0.03), a post hoc statistical power analysis was calculated to 0.65. The inclusion criteria required participants to (a) have been engaged in systematic swimming training for the previous three years, (b) be free from injury, and (c) abstain from medication before, during, and after the experimental procedures. Before data collection, swimmers and their parents or legal guardians were fully informed about the study objectives and experimental procedures. The study received approval from the local institutional review board (approval number: 1749/20-02-2025) and was conducted in accordance with the Declaration of Helsinki for research involving human subjects.

### 2.2. Study Design

**Figure 1:**
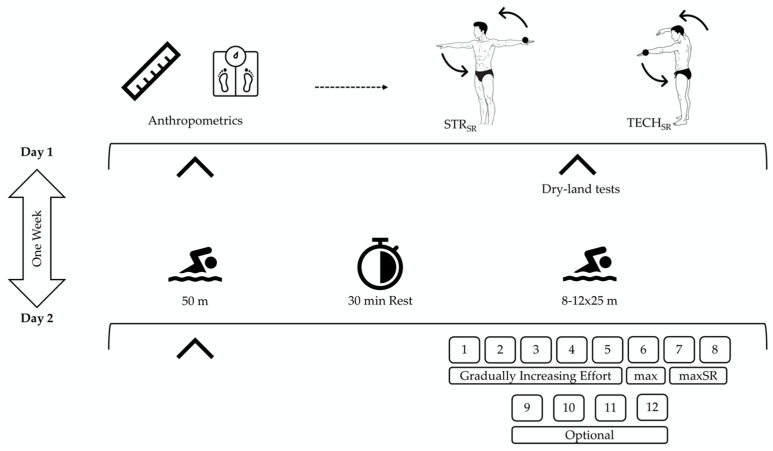
Experimental design of the study. STR_SR_: straight-arm condition, TECH_SR_: bend-arm condition, max: maximum, maxSR: maximum stroke rate.

A one-group repeated-measures design was utilized in the current pilot study. Swimmers with a randomized counterbalanced order were evaluated in two out-of-the-water testing conditions: (i) straight-arm movements (STR_SR_) and (ii) swimming technique simulated bend-arm movements (TECH_SR_). Both conditions required cyclic upper-limb movements as fast as possible. Then, swimmers performed in-water performance tests (Figure 1). All tests were conducted across two sessions, one week apart, and scheduled during the swimmers’ regular Saturday-morning training time. All assessments were conducted at the participants’ habitual training venue, a 50 m indoor swimming pool, with a constant water temperature of 26 °C and humidity of 28–29 °C.

### 2.3. Preliminary Tests and a Familiarization Session

During the first visit, swimmers’ body mass, height (Seca, Hamburg, Germany), and body fat percentage were measured by an experienced investigator following standardized procedures [24]. Following the anthropometric assessments, swimmers were familiarized with the out-of-the-water testing procedures. Subsequently, participants performed two 10 s maximum-effort trials of circular upper-limb movements to assess the maximum movement frequency. The first trial was performed with the upper limbs fully extended (straight-arm condition; STR_SR_), whereas the second trial involved a simulated swimming-technique pattern with elbow-bend upper limbs (TECH_SR_). The STR_SR_ was intended to reflect the participant’s maximal mechanical movement potential, whereas the TECH_SR_ incorporated technique-related constraints that resemble swimming-specific coordination demands. Together, these conditions provided complementary perspectives on upper-limb movement capacity.

### 2.4. Out of the Water Tests

**Figure 2:**
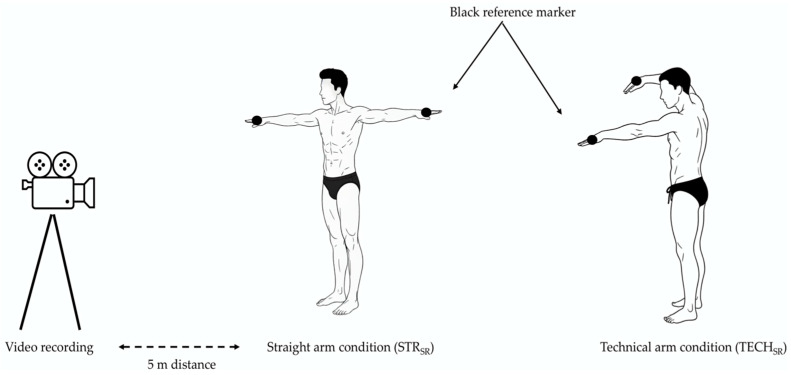
The execution of the swimmer’s arm movement in each condition.

Before STR_SR_ and TECH_SR_ conditions, swimmers performed a standardized shoulder-focused warm-up (e.g., shoulder rotations, upper-limb rotations, and alternating and simultaneous forward and backward arm swings). The upper-limb movement was recorded with a digital video camera (SONY DCR-DVD100, Sony Corp., Tokyo, Japan) mounted on a tripod (SENTIO TR-800, Plesi, Sydney, Australia) and placed vertically to the right side of the swimmer. The camera operated at 50 Hz and was positioned approximately 5 m from the participant at shoulder height to ensure a clear lateral view (Figure 2). A white background with a black line reference marker on the wall was positioned next to the participants to facilitate temporal analysis of limb movement (Figure 2). Video recordings were analyzed using motion analysis software Kinovea (version 0.7.10). Dry-land movement frequency in STR_SR_ and TECH_SR_ conditions was evaluated by dividing 180 by the time required for the right upper-limb wrist to pass the reference marker three consecutive times. Swimmers were instructed to perform all upper-limb movements as fast as possible while maintaining a consistent movement pattern and range of motion across trials. The maximum speed values from the STR_SR_ and TECH_SR_ conditions were used in the statistical analysis.

### 2.5. In-Water Tests

Performance tests were conducted in two sessions, one week apart. During the first in-water testing session, swimmers performed a maximum 50 m front-crawl sprint from a block start. The SR was calculated by measuring the time to complete three stroke cycles during the second 25 m of the 50 m sprint. Stroke length (SL) was determined by the ratio of swimming speed to SR. The product of SL and swimming speed was then used to calculate the stroke index (SI).

**Figure 3:**
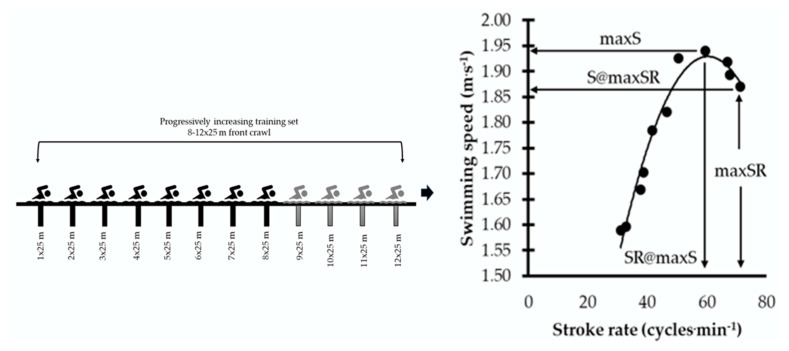
The training set of 8–12 repetitions of 25 m (8–12 × 25 m) front crawl with progressively increasing effort, along with the relationship between swimming speed and stroke rate extracted from the training set. Also, it illustrates how the maximum stroke rate (maxSR), maximum speed (maxS), stroke rate at maximum speed (S@maxSR), and speed at maximum SR (S@maSR) are calculated using the relationship between swimming speed and stroke rate.

One week later, during the second in-water testing session, swimmers performed a training set consisting of 8–12 repetitions of 25 m front crawl (8–12 × 25 m; Figure 3). The protocol’s progressive nature was designed to facilitate the identification of both optimal and maximal stroke-rate responses as speed demands increased. This type of progressive protocol is commonly used in swimming practice to examine the relationship between SR and swimming speed under increasing-intensity conditions, allowing determination of both optimal and maximal SR responses in an ecologically valid setting [9]. Moreover, swimmers were instructed to maintain consistent stroke technique and follow the intended pacing throughout all repetitions. This approach enabled differentiation between performance-related stroke regulation and absolute stroke-rate capacity. Swimming speed was progressively increased across repetitions, with swimmers instructed to gradually raise SR and reach maximum speed between the sixth and eighth repetition. Reaching the maximum possible SR was the purpose during the eighth to the twelfth repetition. A passive rest interval of 2–5 min was allowed between repetitions, and each 25 m repetition commenced with a push start from a wall. Swimming speed (S) was computed as the ratio of distance to time. An experienced timekeeper recorded the time to complete each 25 m effort.

From the 8–12 × 25 m test, the maximum speed (maxS) and maximum stroke rate (maxSR) were identified for each swimmer (see Figure 3). In addition, the SR corresponding to maximum speed (SR@maxS) and the speed corresponding to maximum SR (S@maxSR) were recorded. SL and SI were also computed for both maximum speed (SL@maxS, SI@maxS) and maximum SR (SL@maxSR, SI@maxSR). The ratio of the maximum SR measured in the water during the 8–12 × 25 m test to the movement frequency measured out of the water in both conditions (maxSR/STR_SR_ and maxSR/TECH_SR_) was calculated, expressed as percentage values, and used for statistical analysis. Prior to all swimming performance tests, swimmers completed a standardized warm-up protocol consisting of 400 m of self-selected stroke, followed by 2 × 50 m front crawl at moderate intensity (approximately 160 beats·min^−1^), and 2 × 12.5 m front crawl at maximum effort.

### 2.6. Statistical Analysis

Normal distribution of the data was tested using the Kolmogorov–Smirnov test. Pearson correlation was used to examine relationships between STR_SR_ and TECH_SR_, with the swimming speed and kinematic variables calculated during the 50 and 8–12 × 25 m tests. Moreover, Pearson correlation was used to examine relationships between the percentage values of the ratios maxSR/STR_SR_ and maxSR/TECH_SR_ with the swimming speed and kinematic variables calculated during the 8–12 × 25 m test. The 95% confidence intervals were also calculated for each variable. The Intraclass Correlation Coefficient (ICC) with a one-way random-effects model was used to assess reliability. Data are presented as mean ± SD. Statistical significance was established at *p* ≤ 0.05.

## 3. Results

### 3.1. Performance and Kinematic Variables in Swimming and Land Tests

Land-movement frequency was higher in STR_SR_ compared to TECH_SR_ (mean ± SD [95% CI], 104.5 ± 12.1 [97.0, 111.9] vs. 75.4 ± 6.8 [71.2, 79.69], *p* < 0.05). The mean values of the kinematic variables calculated in the 50 m trial and the 8–12 × 25 m set are presented in Table 2. The maxSR achieved at the 8–12 × 25 m test was higher compared to SR@maxS (*p* < 0.01) and the SR_50_ (*p* < 0.01; Table 2).

### 3.2. Swimming Stroke Rate and Land-Movement Frequency Ratio

**Figure 4:**
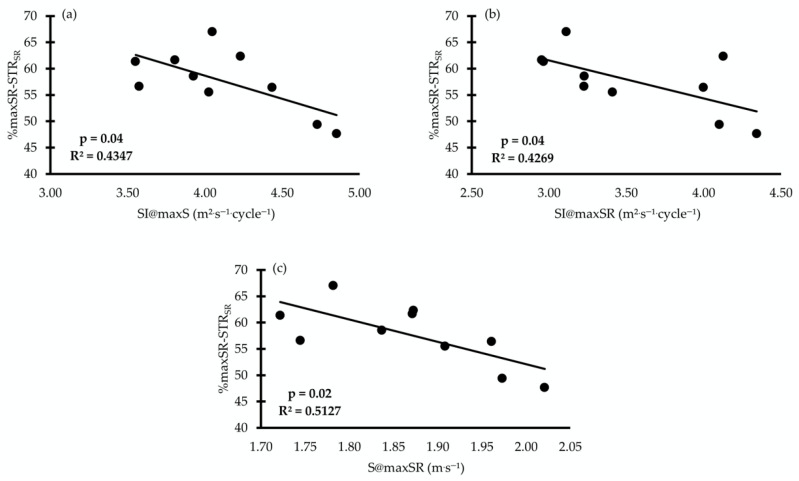
Correlations between the ratio of maximum stroke rate to land-movement frequency during the straight-arm condition (%maxSR/STR_SR_) with the swimmer’s stroke index at maximum speed (SI@maxS, (**a**)), stroke index at maximum stroke rate (SI@maxSR, (**b**)), and speed at maximum stroke rate (S@maxSR, (**c**)).

**Figure 5:**
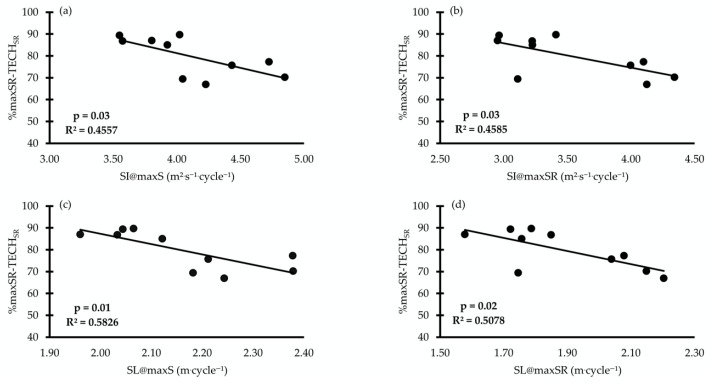
Correlations between the ratio of maximum stroke rate to the land-movement frequency during the bend-arm condition (%maxSR/TECH_SR_) with the swimmer’s stroke index at maximum speed (SI@maxS, (**a**)), stroke index at maximum stroke rate (SI@maxSR, (**b**)), stroke length at maximum speed (SL@maxS, (**c**)), and stroke length at maximum stroke rate (SL@maxSR, (**d**)).

When the ratio of the SR to land-movement frequency was calculated and expressed as the percentage differences in %maxSR/STR_SR_ and %maxSR/TECH_SR_, a relationship was found between %maxSR/STR_SR_, SI@maxS, S@maxSR, and SI@maxSR (r = −0.65 to −0.72, *p* = 0.01, Figure 4). The %maxSR/TECH_SR_ was related to SI@maxS, SI@maxSR, SL@maxS, and SL@maxSR (r = −0.67 to −0.71, *p* = 0.01, Figure 5).

### 3.3. Relation of Movement Frequency to Kinematic Variables

The land-movement frequency in STR_SR_ and TECH_SR_ conditions was not related to the kinematic variables of the 50 m sprint (r = 0.04 to 0.39; *p* = 0.09 to 0.90). Moreover, land-movement frequency in the STR_SR_ and TECH_SR_ conditions was not related to the maxSR, maxS, SR@maxS, SL@maxS, SI@maxS, S@maxSR, SL@maxSR, and SI@maxSR calculated from the 8–12 × 25 m training set (r = 0.01 to 0.56; *p* = 0.09 to 0.99).

## 4. Discussion

The present study examined the relationship between upper-limb movement frequency measured in dry-land with SR measured in a 50 m sprint, as well as kinematic variables during the 8–12 × 25 m test. The main findings indicate that dry-land upper-limb movement frequency was not directly related to in-water SR, 50 m swimming speed, or 8–12 × 25 m test kinematics. However, swimmers who used a lower percentage of their maximum dry-land movement frequency during the 8–12 × 25 test achieved higher swimming speed. This is the first study indicating that the ratios of maximum stroke rate (maxSR) and the frequency of cyclic movements out of the water (STR_SR_ and TECH_SR_) provide some evidence for the transfer of in-water stroke mechanics from land to water.

From a broader motor-control perspective, these findings may be interpreted through the lens of the speed–accuracy trade-off principle [19], which holds that increasing movement frequency may compromise coordination stability and force precision. In cyclic locomotor tasks, athletes rarely operate at their maximum possible frequency; instead, they self-organize toward an optimal frequency that balances mechanical output and coordinative control, as shown in cycling cadence selection and rowing stroke-rate regulation [20,25]. Accordingly, stroke-rate regulation in swimming may reflect a similar optimization process rather than a direct expression of maximal limb turnover capacity.

The observed inverse relationships (see Figure 3 and Figure 4) between the SR and out-of-the-water movement frequency ratios may be connected to the technical constraints that underpin maximum swimming performance. Specifically, SL and SI highlight the importance of dry-land movement frequency. The observed inverse relationship suggests that as maxSR approaches the dry-land STRSR, SI and SL decline measurably. This implies that a swimmer’s ability to convert raw dry-land power into aquatic propulsion is constrained by propulsive efficiency, which often decreases as stroke frequency exceeds a certain threshold [17].

In addition, the relationship between the %maxSR/TECH_SR_ ratio and SL@maxSR indicates that dry-land movement frequency sets a functional ceiling for in-water coordination. When the gap between in-water turnover and out-of-water technical capacity narrows, the swimmer likely loses distance per stroke to maintain tempo. These findings align with the perspective that dry-land power metrics must be balanced with aquatic technical specificity to optimize the stroke index [4]. Consequently, the data suggest that superior swimming economy is found in athletes who maintain a high maxSR without approaching their absolute out-of-water mechanical limits.

Although movement frequency achieved on land was substantially higher than SR in water, no direct association was observed between dry-land and SR measures. This discrepancy can be attributed to markedly different mechanical environments, as water density and drag impose constraints on limb acceleration, coordination, and force application that are absent in air [18]. Consequently, maximum limb turnover on land does not necessarily translate into higher effective stroke rates in water, where propulsion efficiency, stroke timing, and continuity of force production are critical determinants of performance [8,11].

The close correspondence between stroke rate at maximum speed and maximum stroke rate further suggests that swimmers approach their optimal stroke-rate range when performing at maximum speed. However, the most notable finding was that faster swimmers exhibited a larger gap between dry-land speed of movement and in-water SR. This observation implies the presence of a neuromuscular “reserve” in speed of movement that is not fully exploited during swimming, potentially allowing swimmers to operate at a stroke rate that maximizes propulsion while avoiding excessive drag and coordination breakdown [5]. A comparable concept has been described in sprint running, where maximal stride frequency exceeds that at optimal velocity, indicating a functional stride-frequency reserve [21]. Similarly, performance research has shown that excessive step frequency may reduce the application of effective force despite higher limb turnover [26]. These findings support the notion that superior swimmers may benefit not by maximizing SR per se, but by operating below their absolute neuromuscular ceiling while preserving propulsive effectiveness.

From a mechanistic perspective, this reserve may reflect superior neuromuscular capacity combined with refined motor control, enabling swimmers to modulate stroke rate to preserve stroke length, propulsive continuity, and inter-limb coordination. Operating below maximum limb turnover may facilitate more effective force application against the water, reduce non-propulsive phases, and limit energy dissipation due to excessive intra-cycle speed fluctuations [27]. From a coordination-dynamics perspective, increasing movement frequency beyond an optimal threshold may destabilize inter-limb timing and increase intra-cycle velocity fluctuations, both of which are known to amplify hydrodynamic drag [17]. The dynamic systems framework further suggests that higher frequencies may shift coordination patterns or reduce temporal stability, potentially explaining the observed reductions in SL and SI as swimmers approach their limits of dry-land movement. The strong relationships between SI@maxSR and SI@maxS and between %maxSR/STR_SR_ and %maxSR/TECH_SR_ support the aforementioned mechanisms.

Collectively, these findings indicate that superior swimming performance may be underpinned by the ability to regulate SR strategically, rather than by maximizing limb turnover. Assessing the relationship between dry-land movement capacity and in-water stroke expression may therefore provide valuable insights into individual SR regulation strategies and efficiency profiles. While the present study is limited by its small and homogeneous sample and by the exclusive focus on upper-limb movements, the results highlight the importance of considering neuromuscular potential, biomechanical constraints, and coordination demands when interpreting SR behavior in competitive swimming.

Practically, the relationship between land-based movement potential and in-water stroke expression may serve as a useful profiling tool for coaches. Monitoring this relationship could help distinguish swimmers who rely primarily on raw movement speed from those who exhibit more efficient, coordination-driven performance, a distinction emphasized in age-group and elite performance models [6,15]. Although a definitive threshold cannot be determined from the current data, the observed ratio between dryland movement frequency and in-water SR may provide practical reference points for analyzing individual stroke regulation and efficiency profiles. In practical terms, this method can help coaches determine whether training should focus on developing neuromuscular speed or on technical stroke adjustments, depending on each swimmer’s profile. Future studies should explore whether this relationship varies across different age groups, performance levels, and event specializations. Additionally, intervention-based research examining the impact of targeted dry-land speed training on in-water stroke regulation would further clarify the transferability of neuromuscular adaptations.

Despite these perspectives, the relatively small sample size should be acknowledged as a limitation, and therefore, the present findings should be considered preliminary and interpreted with caution. Nevertheless, the use of a well-trained, homogeneous sample, along with a repeated-measures design, strengthens the study’s internal validity.

## 5. Conclusions

The present findings suggest that upper-limb movement frequency measured out of the water does not directly predict in-water SR or swimming performance in adolescent male swimmers. Instead, performance advantages appear to be associated with the ability to regulate SR in response to aquatic constraints, rather than with maximum limb turnover capacity per se. Swimmers who exhibited a larger difference between dry-land movement potential and in-water maxSR achieved higher velocities and demonstrated superior stroke efficiency, indicating a more effective utilization of neuromuscular capacity under high-swimming-speed-demanding conditions. These results highlight the importance of stroke rate modulation and efficiency-oriented movement strategies in sprint swimming. From a performance analysis perspective, the relationship between dry-land and in-water stroke rates may serve as a meaningful indicator of technical proficiency and neuromuscular control, offering a complementary approach to evaluating individual performance profiles beyond traditional kinematic descriptors. From a practical standpoint, the observed land–water movement frequency gap may serve as an indicator of technical efficiency rather than merely a measure of movement speed. Coaches may use this information to better understand individual stroke-regulation strategies and guide training decisions.

## Figures and Tables

**Table 1:** Anthropometric characteristics and competitive level of the participants.

Variables	(*n* = 10)
Age (years)	15.2 ± 1.1
Body mass (kg)	67.8 ± 6.2
Body height (cm)	179.2 ± 0.1
Body mass index (%)	21.1 ± 1.5
Sitting height (cm)	87.1 ± 0.1
Arm span (cm)	184.1 ± 0.1
Single arm overhead reach (cm)	229.3 ± 0.1
Word aquatic points (50 m front crawl)	548.5 ± 90.4
Swimming training experience (years)	7.4 ± 1.2
Competitive swimming experience (years)	3.4 ± 1.2

**Table 2:** Comparisons of swimmers’ kinematic characteristics between the 50 m maximum trial and the 8–12 × 25 m set. Data reported as mean values and standard deviation (Mean ± SD). The 95% confidence intervals (CI) for each variable are also reported in the table.

Variables		50 m Sprint	8–12 × 25 m Test	
			@maxS	@maxSR
S (m·s^−1^)	Mean ± SD	1.78 ± 0.11	1.90 ± 0.1	1.87 ± 0.1
	95% CI	1.71, 1.85	1.84, 1.96	1.81, 1.93
	*p*	-	<0.01	<0.01
SR (cycles·min^−1^)	Mean ± SD	51.3 ± 4.8	53.1 ± 3.1	59.8 ± 5.5
	95% CI	48.3, 54.3	51.2, 55.0	56.4, 63.2
	*p*	-	0.08	<0.01
SL (m·cycle^−1^)	Mean ± SD	2.1 ± 0.2	2.2 ± 0.1	1.8 ± 0.2
	95% CI	1.9, 2.2	2.1, 2.3	1.7, 2.0
	*p*	-	0.03	<0.01
SI (m^2^·s^−1^·cycle^−1^)	Mean ± SD	3.8 ± 0.6	4.1 ± 0.5	3.6 ± 0.5
	95% CI	3.4, 4.1	3.8, 4.0	3.2, 3.9
	*p*	-	<0.01	0.08

Swimming speed (S), stroke rate (SR), stroke length (SL), stroke index (SI), at maximum speed (@maxS), at maximum stroke rate (@maxSR).

## Data Availability

The data will be made available upon reasonable request to the corresponding author.

## Abbreviations

The following abbreviations are used in this manuscript:

STR_SR_	Straight-arm condition
TECH_SR_	Bend-arm condition
S	Swimming speed
SR	Stroke rate
SL	Stroke length
SI	Stroke index
maxS	Maximum swimming speed
maxSR	Maximum stroke rate
S@maxSR	Swimming speed corresponding to maximum stroke rate
SL@maxSR	Stroke length corresponding to maximum stroke rate
SI@maxSR	Stroke index corresponding to maximum stroke rate
SR@maxS	Stroke rate corresponding to maximum speed
SL@maxS	Stroke length corresponding to maximum speed
SI@maxS	Stroke index corresponding to maximum speed
